# Informal care at old age at home and in nursing homes: determinants and economic value

**DOI:** 10.1007/s10198-023-01601-x

**Published:** 2023-06-10

**Authors:** Quitterie Roquebert, Marianne Tenand

**Affiliations:** 1 Université de Strasbourg, Université de Lorraine, CNRS, BETA, 67000 Strasbourg, France; 2 Erasmus School of Health Policy & Management (ESHPM), Erasmus Centre for Health Economics Rotterdam (EsCHER), Erasmus University Rotterdam (EUR) and the Netherlands Bureau for Economic Policy Analysis (CPB), Rotterdam and The Hague, The Netherlands

**Keywords:** Informal care, Long-term care, Aging, Valuation, Decomposition, D10, I10, J14, I18

## Abstract

This paper provides a comprehensive analysis of informal care receipt by the French individuals aged 60 or older. The literature has focused on the community, leaving informal care in residential care settings in the shadow. We leverage data from a representative survey (CARE) conducted in 2015–2016 on both community-dwelling individuals and nursing home residents. Focusing on the 60+ with activity restrictions, we show that 76% of nursing home residents receive help with the activities of daily living from relatives, against 55% in the community. The number of hours conditional on receipt is yet 3.5 times higher in the community. Informal care represents 186 million hours per month and a value equivalent to 1.1% of GDP at least, care in the community representing 95% of the total. We investigate the determinants of informal care receipt. Using an Oaxaca-type approach, we disentangle between two mechanisms explaining that nursing home residents are more likely to receive informal care, namely the differences in population composition (endowments) and the differences in the association of individual characteristics with informal care (coefficients). Both are found to have a similar contribution. Our results imply that private costs make up for the majority (76%) of the costs associated with long-term care provision once informal care is taken into account. They also highlight that informal care is extremely common for nursing home residents. Existing evidence on the determinants of informal care receipt in the community has, however, limited relevance to understand informal care behaviors in nursing homes.

**Supplementary Information:**

The online version contains supplementary material available at 10.1007/s10198-023-01601-x.

## Introduction

Population aging is associated to an increase of long-term care (LTC) needs and costs. Covering a large range of services, long-term care can be provided either by relatives (informal care) or by professional caregivers (formal care). In France, public policies tend both to encourage the use of formal care services through targeted subsidies (APA program, *Allocation personnalisée d’autonomie*), and to support informal caregivers. The importance of informal caregivers for individuals living in the community is well-acknowledged. In 2015, 81% of French older individuals living at home and receiving care were provided support by their relatives [14]. While the economic literature has extensively studied informal care provided at home, there is limited quantitative evidence on the role that relatives play for nursing home residents [28]. Some studies, mostly qualitative, show that relatives still play a major role in assisting nursing home residents with activities of daily living, on top of providing emotional support [19, 21, 28, 29, 42, 44].

The literature has estimated the economic value of informal care, demonstrating that informal care has a sizable opportunity cost for society despite being provided mostly for free [41, 43]. Such studies estimate the monetary value of informal care either at an individual level, or at an aggregate (e.g. country) level. Many papers focus on informal care provided to specific populations, such as individuals with Alzheimer’s disease [20, 36]. Another trait of this literature is that most research focuses on informal care provided in the community, with a few exceptions [38]. In the case of France, the monetary value of yearly informal care receipt among the 60+ in the community alone was estimated to reach 6.6 billion euros in 2000, or 50% more than the costs of formal home care at the time [35].

This paper tackles the following questions: (i) What is the importance and the monetary value of the informal care for older people in France, when also taking into informal care provided in nursing homes? (ii) What are the differences in informal care receipt across the community-dwelling population and nursing home residents, and (iii) To what extent can such differences across the two settings be explained by differences in population composition, or by differences in how individual characteristics predict informal care receipt?

We leverage a survey representative of the whole 60+ French population, split into a sample representative of the community-dwelling population and a sample representative of individuals permanently residing in a nursing home. This high-quality survey allows us to provide a comprehensive assessment of informal care receipt among older adults, as well as an original comparative perspective across the at-home and nursing home settings.

We provide three sets of results. First, we document the receipt of informal care care at home and in nursing homes. We show that no less than 76% of nursing home residents receive informal support with the activities of daily living, with an average volume of 24 h a month. Second, we estimate the economic value of informal care, taking into account the unexplored economic value of informal care in nursing homes. We use the proxy good method, a partial valuation method, thereby informal care hours received by individuals are valuated using a close market substitute (cost of professional caregivers). Throughout the estimation steps we adopt a conservative approach, so as to retrieve a lower bound for the aggregate monetary value of informal care. We find that the yearly informal care provision represented in 2015/2016 about 24.2 billion current euros (1.1% of GDP) at least. Care provided in nursing homes represent 5% of the aggregate value. Third, we study the determinants of informal care receipt at the extensive margin. Differences across residential settings can be attributed to (i) differences in the characteristics of the two sub-populations (endowment effect) and (ii) differences in the way individual characteristics relate to the probability to receive informal care (coefficient effect), and (iii) their interaction. We use an Oaxaca-type approach, which makes it possible to disentangle between these mechanisms [2, 32]. We find that differences in the composition of the population across the two settings only partially explain differences in informal care provision. It thus points towards different informal care behaviors in each setting for individuals with the same characteristics.

## Data

### CARE survey

We take advantage of the survey Capacités, Aides et REssources des seniors (CARE), a general population survey targeting the French population aged 60 and older. The CARE survey was conducted in 2015–2016 by the statistical division of the Ministry of Health (Drees) to document the living conditions of the 60+, their relationships with their relatives, the limitations they face as well as the human, technical and financial support they receive. The survey consists of two parts: CARE-Ménages (CARE-M, 2015) is devoted to the individuals living in the community, while CARE-Institutions (CARE-I, 2016) surveys older people whose permanent residence is an assisted-living facility or a nursing home.^1^ For the sake of simplicity, we call the population sampled in CARE-I ‘nursing home residents’.

The CARE survey comes with four main advantages for the purpose of our study. First, samples are representative of the 60+ French population not only in terms of socio-demographic characteristics, but also in terms of overall health status. Appendix A provides more information on the sampling procedure. Second, the individuals with poor health were over-sampled so that we are able to work with relatively large sample sizes when focusing on the disabled individuals. Third, the survey has high response rates (88% at the nursing home level and 86% at the respondent level for CARE-I, 73% at the respondent level for CARE-M), meaning that selective non-response in terms of unobservable characteristics can be expected to be limited. Finally, respondents are asked to list up to 10 of their informal caregivers: unlike many previous studies relying on data on the ‘primary caregiver’ only, we are thus able to include all their informal caregivers (identified as such by respondents).

### Study population and sample selection

We focus on individuals with restrictions in the activities of daily living and have thereby a care need: our study population is made of individuals needing assistance with any activity of daily living, either essential (ADL) or instrumental (IADL). ADLs cover grooming, dressing and undressing, using toilets, cutting food, eating and drinking, transferring from a chair and transferring from bed (7 ADL activities). IADLs cover moving in the place where one lives, doing housework, doing administrative tasks, doing grocery shopping, preparing meals, taking medication, using a phone, going outside, using transportation, finding one’s way outside (10 IADL activities). For each of these activities, the respondent has to answer to the question:“Do you perform this activity without any help?” by choosing one of the following items: “1. Yes, without any difficulty; 2. Yes, but with some difficulties; 3.Yes, but with a lot of difficulties; 4. No, I need help”. The respondent is included in our sample if she/he ticks item 2, 3 or 4 for at least one activity (either ADL or IADL). Appendix B.1 provides more details on the distribution of restrictions with each ADL and IADL in reference populations.

In nursing homes, virtually all (99%) individuals do have at least one ADL/IADL limitation. In the community, those with ADLs/IADLs restrictions represent 32% of the population. These individuals are older, more frequently women and living alone, they have more children, compared to individuals living in the community without any activity restrictions.

### Information on informal care receipt and outcome definition

The survey provides a rich set of information on the care received by individuals, provided either by relatives or professionals. Regarding informal care, we observe the number of informal caregivers and for each caregiver, the type of care provided among the following: care for with ADLs/IADLs, financial or material support, moral support. We have information on volume for caregivers dealing with ADLs/IADLs: when an individual declares a caregiver for with ADLs/IADLs, she/he is asked about the volume of care she/he receives from this caregiver. When she/he is not able or willing to estimate a precise number, she/he can select an hour range.

We are interested in the volume of informal care received by individuals: we use the number of hours declared when it is directly available; when not available, we choose the lowest bound of the interval of hours declared by the individual. Only when the interval included zero, we rather choose half the higher bound (0.5 h for a range [0;1] per day, 3.5 h for a range of [0;7] per week, 15 h for a range of [0;30] per month). Individuals may provide the number of hours (or interval) per day, week or month. We express all volumes at the monthly level: daily (resp. weekly) volumes were multiplied by 30 (resp. 4.33). Finally, we compute the total volume of informal care received by the individual by summing the hours provided by all the caregivers. To deal with implausible or extreme values, we censor the volume of hours received at the *caregiver* level, as as is often done in informal care valuation [34, 37]. We set a maximum of 12 h per day, corresponding to the maximum daily duration of work in France. In addition, we censor the care volume at the *respondent* level (that is, the care recipient) to 24 h per day, to make our estimations less sensitive to extreme values.^2^

We focus on informal care that is provided for ADL/IADL activities, for two reasons. First, it can be quantified in hours, while such a quantification is harder for moral support.^3^ As a matter of fact, the CARE survey did not ask respondents to estimate the volume of the moral support they receive. Second, care with ADLs/IADLs may be provided either formally or informally. This provides a rationale for valuing care with ADLs/IADLs using the price of professional care, as will be explained in Sect. Economic valuation of informal care.

### Descriptive statistics

#### General descriptive statistics

Table 1 presents the socio-demographic characteristics of the individuals aged 60 and more and facing ADL and/or IADL limitations while Table 2 focuses on their health characteristics. Differences have been tested using a Student test (resp. $\chi ^2$ test) for continuous or dummy (resp. categorical) variables and they are significant at the 1% level (except for sensory limitations, significance at 5% level).

Individuals living at home are younger and more educated than nursing home residents. Nursing home residents are more frequently women, with limited informal care resources: they are on average more frequently widow, single or divorced, without children nor brother(s) or sister(s) alive. Regarding income^4^ individuals with limited resources are more frequently represented in nursing homes. In terms of education, we observe the highest diploma obtained by the individual, which gives an insight into her/his social status. Missing values are much more frequent for individuals in nursing homes, making the comparison of distributions difficult.

Regarding health characteristics, nursing home residents more frequently suffer from restrictions and limitations. Following the epidemiological literature [3, 17], we distinguish between individual with moderate activity restrictions (IADL only), high activity restrictions (ADL) and severe activity restrictions (ADL including those on minimum independence: going to the toilet, self-feeding, getting up and down). The prevalence is higher in nursing homes for severe activity restrictions, (self-declared) Alzheimer disease and limitations at the cognitive, sensory, suppleness/handling or locomotion/balance level. Surprisingly, the distribution of subjective health indicators show that nursing home residents more frequently declare being in good or very good health, rather good health than at-home individuals.

The presence of proxy respondents answering for the individual during the survey is not negligible. 28.3% of community-dwellers receives help for answering the informal care part of the survey, 44.7% when taking the other modules of the questionnaire into account. These proportions increase to 54.9% and 64.5% for nursing home residents. Both dimensions are correlated (correlation coefficient of 0.77) but in the estimations, they are expected to capture two different aspects. Both give an indication on the health status of the individual but the presence of a proxy respondent on informal care question additionally shows that the caregiving volume results from an estimation provided by a relative who is potentially a caregiver her/himself.

**Table 1:** Descriptive statistics: socio-demographic characteristics Sources: CARE-M (2015), CARE-I (2016)

	(1)	(2)	(3)
	At home	In nursing homes	Entire population
Woman	64.9	74.8	66.0
Age: 60–74	36.3	10.1	33.5
Age: 75–84	35.1	21.7	33.6
Age: 85–89	17.2	27.8	18.4
Age: 90–94	9.0	26.9	11.0
Age $\ge$ 95	2.3	13.6	3.5
Married	49.1	12.9	45.1
Widow	35.0	63.3	38.2
Single or divorced	15.8	23.9	16.7
Children: none	11.6	25.6	13.2
Children: 1	20.4	22.5	20.6
Children: 2	30.5	23.9	29.8
Children: 3 or more	37.5	28.0	36.4
Sister(s) or brother(s) alive	71.0	43.9	68.0
Diploma: none	29.2	27.3	28.9
Diploma: primary education	32.1	30.8	32.0
Diploma: secondary education	30.5	18.4	29.2
Diploma: higher education	7.6	4.3	7.3
Diploma: missing	0.6	19.2	2.6
Income: $\le$ 14,999	33.4	43.5	34.5
Income: 15,000-19,999	25.8	25.1	25.7
Income: 20,000-29,999	26.7	21.3	26.1
Income: $\ge$ 30,000	14.2	10.1	13.7
Observations	6889	3161	10,050

Samples: French 60+ population, with activity restrictions, living at home or in a nursing home

Percentages of population are computed taken into account survey weights. Income in annual current euros. The differences between the sample of individuals living in nursing homes and individuals living at home are all significant at the 1% level (Student test -resp. $\chi ^2$ test- for continuous or dummy -resp. categorical- variables)

**Table 2:** Descriptive statistics: health characteristics Sources: CARE-M (2015), CARE-I (2016)

	(1)	(2)	(3)
	At home	Nursing home	Entire population
Restrictions: IADL only	51.6	14.0	47.4
Restrictions: ADL, except those of minimum independence	44.5	41.6	44.2
Restrictions: ADL on minimum independence	3.9	44.4	8.4
Alzheimer’s Disease	4.0	36.0	7.6
Limitations: cognitive	80.0	92.4	81.4
Limitations: sensory	68.2	75.0	68.9
Limitations: suppleness, handling	85.8	95.6	86.9
Limitations: locomotion, balance	66.4	92.7	69.3
Incontinency	27.1	64.9	31.3
Self-reported chronic disease or health condition	80.2	68.3	78.9
Subjective health: bad or very bad	31.3	35.2	31.7
Subjective health: rather good	46.5	41.5	46.0
Subjective health: good or very good	22.2	22.5	22.2
Subjective health: missing	0.0	0.8	0.1
Proxy on informal care questions	28.3	54.9	31.3
Proxy on other parts of the questionnaire	44.7	64.5	46.9
Observations	6889	3161	10,050

Samples: French 60+ population, with activity restrictions, living at home or in a nursing home

Percentages of population are computed taking into account survey weights. The differences between the sample of individuals living in nursing homes and individuals living at home are all significant at the 1% level (Student test -resp. $\chi ^2$ test- for continuous or dummy -resp. categorical- variables), except for sensory limitations, significant at the 5% level

#### Descriptive statistics on informal care

Table 3 provides descriptive statistics on informal care. Part A focuses on care receipt at the extensive margin. About 60% of the 60+ with activity restrictions living in the community receive some informal care. This share is much higher among nursing home residents, over 80%. Echoing previous findings [10, 28, 39], only a minority of the study population declares receiving material or financial support, while moral support and support with ADLs/IADLs are much more frequent. In the community, about 50% of the 60+ with activity restrictions receive support with ADLs/IADLs, against three out of four of nursing home residents.

**Table 3:** Descriptive statistics: informal care receipt Sources: CARE-M (2015), CARE-I (2016)

	At home		In a nursing home	
	Sample	%	Sample	%
A. Informal care receipt–extensive margin				
Study population (individuals with ADL/IADL limitations)	6889	100	3161	100
Receives				
Any informal support	4819	57.9	2581	81.3
Moral support	2436	26.2	2433	76.6
Financial support	395	4.3	398	12.9
Support with one ADL at least	1608	13.9	576	18
Support with one IADL at least	4593	54.3	2403	75.8
Support with one ADL or IADL at least	4649	55.4	2422	76.5
B. Informal care receipt for–intensive margin				
Among recipients of informal care with ADL/IADL				
Declares a positive volume of care	4425	87.8	2,184	90.4
All caregivers have missing volume	224	12.2	238	9.5
Total	4649	100	2422	100
Declares a positive volume and have at least:				
One caregiver with volume provided in a range	1294	30.8	477	19.4
One caregiver with missing volume	0	0	228	9
Average number of caregivers	1.42		1.51	
Average share with volume provided directly	72%		75%	
Average share with volume provided in a range	28%		17%	
Average share with volume missing	0		8%	
Among recipients with positive volume of care with ADL/IADL, in hours/month				
Mean	78.9		23.8	
Standard deviation	109.2		53.5	
Skewness	2.4		7.7	
Maximum	720		720	

Samples: French 60+ population, with activity restrictions, living at home or in an institution

Percentages of population are computed taking into account survey weights

Part B of Table 3 focuses on individuals receiving informal care with ADLs/IADLs. Among the 4,649 (resp. 2,422) survey respondents at home (resp. in a nursing home) who declare being helped with ADLs/IADLs, around 90% report a volume of care for at least one of their informal caregivers. The remaining respondents have either not provided any information on caregivers or have not been able to quantify the care provided for any of his/her caregivers. 30.8% of individuals at home provided a range of volume for a least one caregiver (19.4% in nursing home), while 9.0% of individuals in nursing home have at least one caregiver with missing volume.

The average number of caregivers is about 1.42. In general, individuals have been able to provide directly the value of the volume provided, for about 3/4 of their caregivers on average (72% at home, 75 % in nursing homes). When it is not the case, individuals living at home have systematically provided a range for the volume (28% of caregivers on average), while individuals in nursing home have either provided a range (17% of caregivers on average) or declared no volume (8% of caregivers). There is no significant correlation between respondent/caregivers characteristics and the probability to have a missing or bounded volume (estimations available upon request).

The absence of information on volume for some caregivers implies that care volumes at the respondent level are under-estimated. It affects 12.2% of the community-dwellers, and 18.5% (9.5 + 9) of nursing home residents. Under-estimation comes also into play when the care volume is provided in certain ranges, given that we impute the care volume as the lower bound of the interval. Under-estimation would thus happen for a maximum of 30.8% of the at-home population and 19.4% of nursing home residents. All in all, our measure of care volume is expected to be a lower bound for the true care volume received for 43% of the community-dwellers receiving support with ADLs/IADLs, and 38.1% of those in a nursing home.

Among individuals with a positive volume of informal care available, the average volume of care received at home is estimated to be of 78.9 h/month, over 3.5 times higher than for nursing home residents (23.8 h/month). There is ample inter-individual variation in care receipt at the intensive margin: the distribution of volumes is widely spread and heavily skewed. This pattern is observed in both settings, but to a higher extent in the community.

**Fig. 1:**
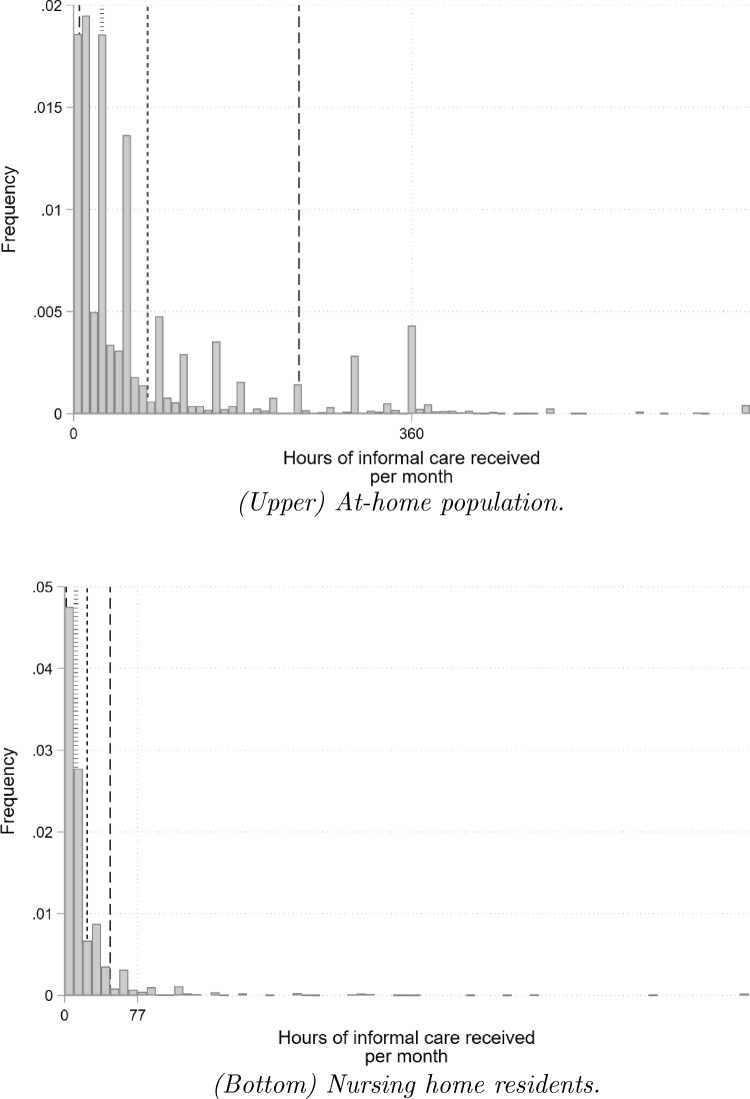
Distribution of the number of informal care hours received, per month, among recipients. Samples: French 60+ population, with activity restrictions, living at home (left) or in an nursing home (right), receiving informal care for the activities of daily living. Statistics are weighted using the survey weights. The vertical continuous and dotted lines correspond to the mean and the median of the number of hours received respectively, computed in each population separately. The dashed lines correspond to the 10^th^ and 90^th^ percentiles Sources: CARE-M (2015), CARE-I (2016)

Figure 1 plots the distribution of care hours among those reporting a positive volume. Note that the distribution exhibits a series of spikes, induced by the conversion of (rounded) hours of care receipt per day or per week into a monthly value.

Overall, these statistics reflect three interesting patterns. First, informal care receipt is extremely common among nursing home residents, although the empirical economic literature has not attached much attention to it [28]. Second, at the intensive margin, informal care is higher in the community than in nursing homes, presumably reflecting the fact that professional care is more prominent in nursing homes than at home. Finally, individual heterogeneity with respect to informal care receipt is higher among individuals living in the community, possibly because of their higher heterogeneity in terms of health and formal support.

## Methods

### Economic valuation of informal care

In a first step, we evaluate the economic value of informal care provided to the 60+ with activity restrictions in France (both at home and in nursing homes), by computing a monetary equivalent of the informal care hours they receive. We opt for a revealed preference method, the proxy good method, for two main reasons. First, it does not require labor market information on the caregivers, while the opportunity cost method would require to know the individual wage’s rate, or estimate a reservation wage for individuals not in the labor market. Second, it is in line with the societal perspective we adopt, as it provides an estimate of the minimum cost that society would incur if informal caregivers would need to be replaced by formal caregivers [41]. This approach makes it therefore possible to compare the monetary value of informal care with private and public spending on formal long-term care.

In this valuation exercise, we restrict our attention to caregivers providing care for ADL/IADL activities and use the volume at the respondent level, computed as explained previously (Sect. Information on informal care receipt and outcome definition). The hours then are valuated using the hourly labor cost for a worker employed at the minimum wage.^5^ In 2015, the hourly labor cost corresponding to this minimum wage amounted to $\text{\EUR}$10.84.^6^ The idea is to measure the monetary equivalent of informal care hours received, using the cost it would represent if it were provided by formal caregivers.

### Econometric approach: informal care determinants and differences across residential settings

In a second step, we examine the difference in the average receipt of informal care in nursing home versus at home. It can be explained as a combination of (a) differences in the composition of the two sub-populations, and (b) differences in how individual or family characteristics influence the probability to receive informal care.

To explore these channels, we use the framework by Oaxaca [32]. It aims at explaining outcome quantitative differences across two populations in statistical terms. The approach needs not be causal [33], and should therefore not be interpreted as such. It has been widely used in the study of inequalities in health and health care use. In the field of long-term care, Bakx and coauthors [2] use a similar decomposition to study the differences in informal care and formal care receipt between Germany and the Netherlands.^7^

#### Informal care receipt as a function of observable characteristics

We denote $y_i$ the informal care received by individual *i* at extensive margin (0/1). For nursing home residents, we assume that $y_i$ can be expressed as:

1 $$y_i = \beta ^{k} X_i + u_i^{k}$$

where $X_i = (1,x^1_i,...,x^J_i)$ is a vector including *J* covariates and *k* is the setting in which the individual lives ($k = inst$ or $k = home$). $\beta ^{k}$ a vector of parameters specific to the setting, including an intercept. $u_i^{k}$ captures the unobserved determinants of informal care receipt in the setting *k*.

#### Decomposing the gap in informal care receipt: endowment versus coefficient differences

Using Equation (1), we express mean informal care receipt for individuals living in the community, denoted $\bar{y}^{home}$, as the product of the parameter estimates ${\hat{\beta }}$ and the population average of covariates for community-dwelling residents, denoted $\bar{X}^{home}$: $\bar{y}^{home} = {\hat{\beta }}^{home}\bar{X}^{home}$. Similarly, for nursing home residents: $\bar{y}^{inst} = {\hat{\beta }}^{inst}\bar{X}^{inst}$. We can then express the gap in mean informal care receipt across the two settings as:

2 $$\begin{aligned} \bar{y}^{inst} - \bar{y}^{home}&= {\hat{\beta }}^{inst} \bar{X}^{inst} - {\hat{\beta }}^{home} \bar{X}^{home} \nonumber \\&= \underbrace{{\hat{\beta }}^{home} \Delta X }_{E} + \underbrace{\Delta \beta \bar{X}^{home}}_{C} + \underbrace{\Delta \beta \Delta X}_{CE} \end{aligned}$$

where:

$$\begin{aligned} {\hat{\beta }}^{home} \Delta X&= {\hat{\beta }}^{home} \big ( \bar{X}^{inst} - \bar{X}^{home} \big ) \\&= \sum _{j=1}^J {\hat{\beta }}_j^{home} \big ( \bar{x}^{j,inst} - \bar{x}^{j,home} \big ) \\ \Delta \beta \bar{X}^{home}&= \big ( {\hat{\beta }}^{inst} - {\hat{\beta }}^{home} \big ) \bar{X}^{home} \\&= \sum _{j=1}^J \big ( {\hat{\beta }}_j^{inst} - {\hat{\beta }}_j^{home} \big ) \bar{x}^{j,home} \\ \Delta \beta \Delta X&= \big ( {\hat{\beta }}^{inst} - {\hat{\beta }}^{home} \big ) \big ( \bar{X}^{inst} - \bar{X}^{home} \big ) \\&= \sum _{j=1}^J (\bar{x}^{j,inst} - \bar{x}^{j,home})({\hat{\beta }}^{inst}_j - {\hat{\beta }}^{home}_j) \end{aligned}$$

Equation (2) indicates that the gap in mean informal care receipt across the two populations can be decomposed as the sum of the gap in individual characteristics (or gap in *endowments*, denoted *E*), the gap in the partial correlations between individual characteristics and informal care receipt (or gap in *coefficients*, denoted *C*) and the interaction of the two (*CE*). In the general case, the interaction term does not correspond to the product of endowment and coefficient terms ($CE \ne C \times E$).

The interpretation of the endowment term and the composition term goes as follows. If the composition of the community-dwelling and the nursing home populations were exactly the same, the higher probability of informal care receipt among nursing home residents would be entirely attributable to the fact that individual characteristics play differently in the determination of informal care receipt at home and in nursing homes. We would have: $E=0$, $CE=0$ and $\bar{y}^{inst} - \bar{y}^{home} = C$. In the polar case, all observable characteristics would display the same partial correlation with informal care receipt in nursing homes and in the community; the higher probability of informal care receipt would then be explained entirely by the differences in the composition of the population across the two residential settings: $C=0$, $CE=0$ and $\bar{y}^{inst} - \bar{y}^{home} = E$. Between these two polar cases, we may expect the gap in informal care receipt to be attributable to both composition differences and coefficient differences, and to their interplay. The interaction term could be regarded as the differential effect of a difference in endowments (resp. coefficients) when coefficients (resp. endowments) differ [18]. Appendix C provides more details.

#### Implementation

Empirically, the decomposition of Equation (2) is achieved by estimating Equation (1) by Ordinary Least Squares (OLS), and by estimating the empirical mean of each covariate, for at-home individuals and nursing home residents separately. The survey weights are used both in the OLS regressions and to compute the population mean of covariates. To take into account potential correlations of disturbances across individuals living in the same nursing home, we estimate standard errors clustered at the nursing home level for ${\hat{\beta }}^{inst}$. Given that each of the at-home respondents belongs to a distinct household, we estimate unclustered standard errors for estimates ${\hat{\beta }}^{home}$. Econometric analyses were conducted using the econometric software Stata (16.1). We use the user command decompose [27], as well the command oaxaca, which further provides the standard errors.

As we focus on the extensive margin of informal care, the OLS model amounts to a linear probability model. The Oaxaca decomposition then sheds light on the individual characteristics associated with the probability of informal care receipt and how they differ across both residential settings.

## Results

### Economic valuation of informal care

We first value the informal care received by older people to help them with the activities of daily living, including both care in nursing homes and in the community. Table 4 presents the result of the valuation. More than half of the community-dwellers and over 3/4 of nursing home residents receive informal care. A nursing home resident receives 23.8 h/month on average conditional on care receipt, against 78.9 h/month in the community. To derive the *unconditional* mean care hours in both settings, we multiply the *conditional* mean by the weighted share of respondents who provided information on the *volume* of care they receive (in Column (2)), which is about 7 percentage points lower than the share of the population reporting care *receipt*. In this way, we use the survey responses conservatively, so as to construct a lower bound for the monetary value of informal care. Unconditional mean hours are about three times higher on average in the community than among nursing home residents (equal to Column (2) multiplied by Column (3), i.e. 43 h against 15 h, *not displayed in the table*).

We then scale up these individual-level estimates by the population size (which is estimated using the survey weights): 4.6 million community-dwelling 60+ with activity restrictions on one side, and about 10 times less nursing home residents on the other side (570 thousands; Column (4)). The community-dwellers are estimated to receive 176 million hours of care with ADLs/IADLs from their relatives every month, while the hours provided to nursing home residents sum up to (only) 9.4 millions. Plugging in an hourly value of care of $\text{\EUR}$10.84, we get to a total yearly monetary value of about 24.2 billion euros (Column (6)). The value of informal care in nursing homes represents over 5% of the total value.

**Table 4:** Economic valuation of informal care in 2015/2016

	Receives informal care (%)	Provides volume information (%)	Mean number of care hours^a^	Weighted population (in thousands)	Total hours (in M.hours /month)^b^	Monetary value (yearly, M$\text{\EUR}$)^c^
	(1)	(2)	(3)	(4)	(5)=	(6)
					$$(2)\times (3)\times (4)$$	
At home	55.4	48.6	78.9	4600	176.4	22,945
In nursing homes	76.5	69.2	23.8	570	9.4	1221
Total	57.7		70.6	5170	185.8	24,166

^a^Mean number of hours of informal care conditional on receiving any. ^b^Total hours expressed in million hours per month. ^c^Monetary value expressed in million euros

Statistics in Columns (1) to (3) are weighted using the CARE survey weights. Weighted population in Column (4) is obtained using survey weights. Each hour is valuated using the hourly labor cost corresponding to the minimum wage in France in January 2016 ($\text{\EUR}$10.84)

### Determinants of informal care at home versus in nursing homes

**Fig. 2:**
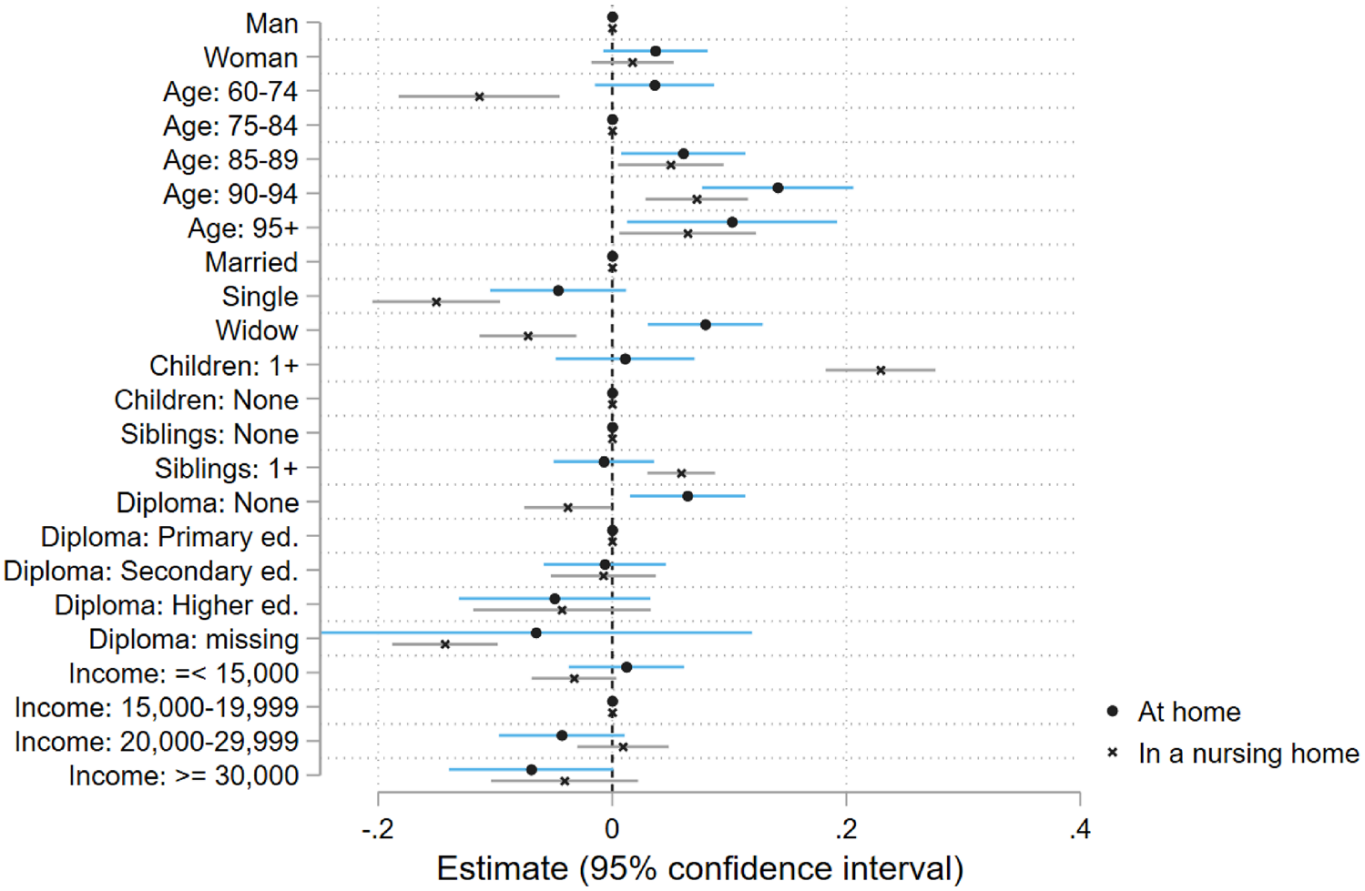
Socio-demographic determinants of informal care (extensive margin). Samples: French 60+ population, with activity restrictions, living at home or in an nursing home. Coefficients corresponding to the OLS regressions of the probability to receive informal care on socio-demographic, limitations and proxy variables Sources: CARE-M (2015), CARE-I (2016)

**Fig. 3:**
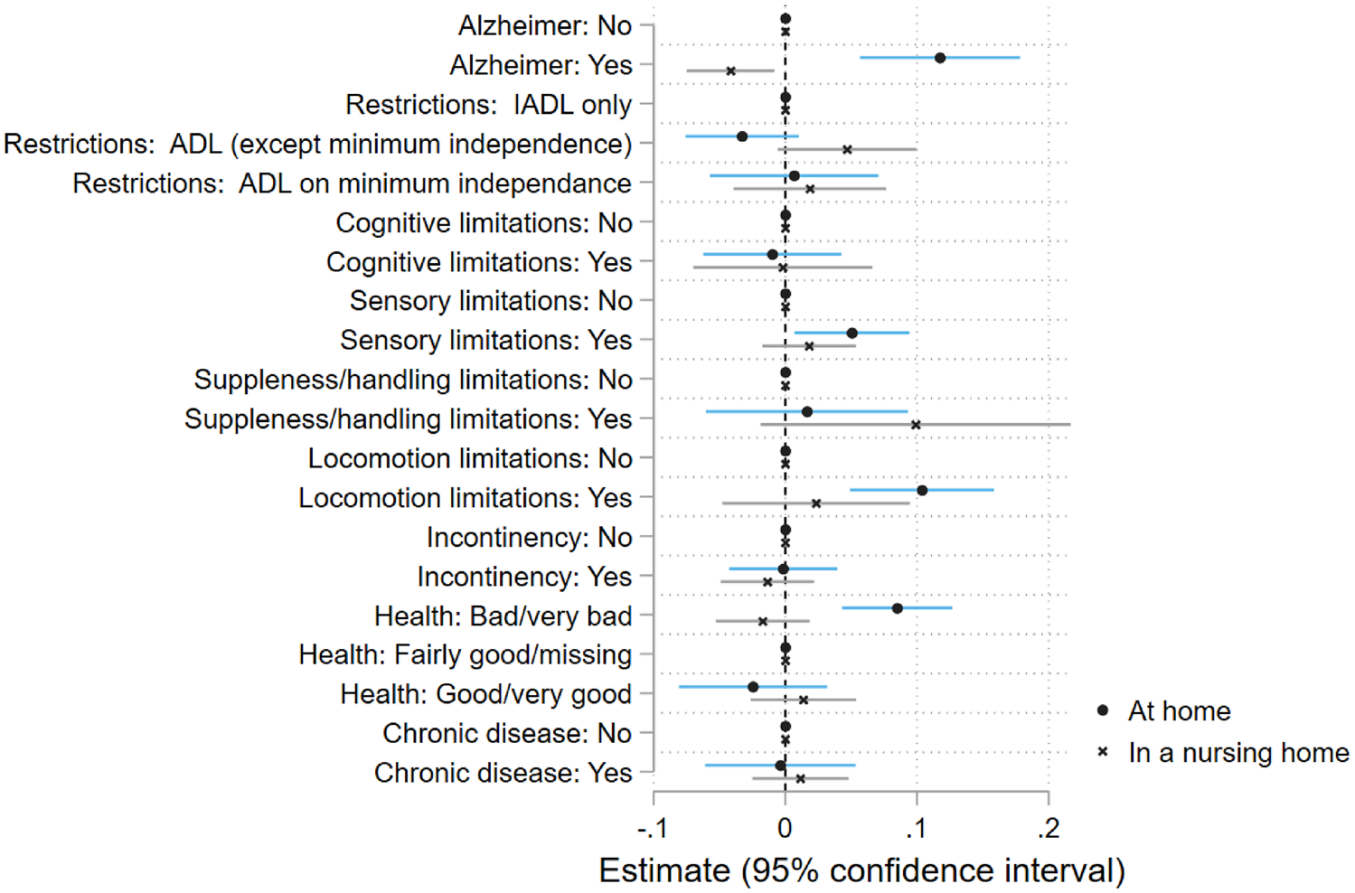
Limitations as determinants of informal care (extensive margin). Samples: French 60+ population, with activity restrictions, living at home or in an nursing home. Coefficients corresponding to the OLS regressions of the probability to receive informal care on socio-demographic, limitations and proxy variables CARE-M (2015), CARE-I (2016)

Figures 2 and 3 show the OLS estimates for the population in the community and for nursing home residents.^8^ Looking at the socio-demographic characteristics (Fig. 2), gender is not significantly associated with the probability of receiving informal care. In both settings there is an age gradient, which is somehow more marked at home: in the community, being aged 90 to 94 years old increases the probability of receiving informal care by over 14 percentage points, against 7 percentage points in nursing homes, relative to the reference category (aged 75 to 84). Interestingly, being aged less than 75 decreases the probability of receiving informal care with ADLs/IADLs in nursing homes (by 12 percentage points), but not in the community. Among the population of people under age 75, a nursing home admission is relatively rare: young nursing home entrants might have unobserved characteristics that would explain their lower average informal care receipt (e.g. absence of relationships with their relatives).

The probability of receiving informal care is sensitive to the availability of potential caregivers, especially for nursing home residents. Compared with individuals with a partner, those who are single or divorced have a lower probability of receiving informal care in both settings. Being a widow, having no children or no siblings decreases the probability of care receipt among nursing home residents. By contrast, these variables have no effect for individuals in the community.

In the community, there is an education gradient: the lower education, the higher the probability of informal care receipt; in nursing homes, having no diploma is associated to a lower probability to receive informal care. The difference across the two settings should however be interpreted with caution since diploma is missing for a substantial share of nursing home residents and missing observations are strongly, negatively associated with informal care receipt in nursing homes. Higher income is associated with a slightly lower probability to receive informal care for individuals living at home.

Regarding health and functional status (Fig. 3), we observe overall no significant effect of the disability group both in the community and in nursing homes. Except that Alzheimer’s disease is strongly positively associated with care receipt in the community (+11.2 percentage points), but decreases the probability of informal care receipt among nursing home residents. Patients with dementia might require constant surveillance, which is provided by the residential structure to nursing home residents, while at home the costs of the round-the-clock presence of a professional caregiver cannot usually be borne. Sensory limitations and limitations with locomotion and balance increase informal care receipt at home, while each type of limitations has no detectable effect on care receipt among nursing home residents. The very high proportion of nursing home residents with such limitations (cf. Table 2, Column (2)) might explain the low statistical precision on these variables. A poor subjective health is associated with higher informal care receipt at home but not in nursing homes, relative to reporting a fairly good health. Finally, Appendix E presents results on variables related to proxy responses.

Summing up, in nursing homes, the existence of potential caregivers is the major correlate of informal care receipt. In the community, care receipt is much more related to health characteristics than in nursing homes.

### Decomposition of the gap in informal care receipt across residential settings

We now turn to the baseline decomposition of the gap in informal care receipt across the two residential settings (Table 5).

**Table 5:** Decomposition of the gap in informal care receipt across residential settings Sources: CARE-M (2015), CARE-I (2016)

	Point estimate	Standard error	95% confidence interval
[*a*] Mean care receipt in nursing homes	0.765${*}{*}{*}$	(0.008)	[0.748; 0.781]
[*b*] Mean care receipt at home	0.554${*}{*}{*}$	(0.010)	[0.532; 0.575]
[*c*] Difference in means	0.211${*}{*}{*}$	(0.013)	[0.184; 0.237]
$$=[a]-[b] =[d]+[e]+[f]$$			
[*d*] Contribution of endowments (E)	0.216${*}{*}{*}$	(0.023)	[0.169; 0.261]
[*e*] Contribution of coefficients (C)	0.200${*}{*}{*}$	(0.021)	[0.156; 0.242]
[*f*] Contribution of interaction (CE)	$-$0.204${*}{*}{*}$	(0.029)	$[ -0.261;-0.142]$

Samples: French 60+ population, with activity restrictions, living at home or in a nursing home

Computations made using the Stata command oaxaca. Statistical significance: ^∗^ $p<0.10$, ^∗∗^ $p<0.05$, ^∗∗∗^ $p<0.01$

Would there be only differences in population composition between the community and nursing homes, we would predict the probability of informal care receipt to be 21 percentage points higher in nursing homes than in the community ($E=0.216$, row [*d*]). The positive contribution reflects the fact that, on balance, the characteristics positively associated with care receipt at home are more prevalent in nursing homes than at home (i.e. when $\beta _j^{home}>0$ and $\bar{x}^{j,inst}>\bar{x}^{j,home}$), and/or that the characteristics negatively associated with care receipt at home are less prevalent in nursing homes than at home (i.e. when $\beta _j^{home}<0$ and $\bar{x}^{j,inst}<\bar{x}^{j,home}$). For instance, an older age is positively correlated to informal care and nursing home residents are older on average; conversely, having a higher education diploma is negatively correlated to informal care receipt and is less frequent in nursing homes.

Would there be only differences in how individual characteristics play on informal care receipt, the share of nursing home residents receiving informal care would be 20 percentage points higher than the share of at-home disabled older people receiving such care ($C=0.200$, row [*e*]). The positive sign indicates that, on balance, the partial correlation between individual characteristics and care receipt is smaller in nursing homes than at home (i.e. $\beta _j^{inst}<\beta _j^{home}$). This is the case for most disability and health variables (see Fig. 3).

When summed up over all covariates, the interaction between the gap in the mean value of a given characteristic and the gap in how it associates to informal care receipt contributes to reducing the gap between the two settings, by 20.4 percentage points ($CE=-0.204$, row [*f*]).

## Discussion

### The value of informal care: alternative values and comparison with available estimates

Our estimation of the value of informal care is intended to be a lower bound, for at least two reasons. First, we made conservative choices when calculating the volume of informal care. In particular, we do not impute care volume for respondents who report being helped with ADLs/IADLs but do not provide *any* information on the volume of care received. Similarly, for caregivers for whom care hours are reported in a range, we generally compute the care volume using the lower bound.

Second, we value each hour of informal care use the unit labor cost that corresponds to the minimum that can legally be paid. Care workers may be paid a higher price. With the proxy good method, the average market wage is often used to value informal care [20, 22, 36, 38]. In France however, formal home care workers are typically employed at, or close to the minimum wage [1]. Collective agreements in the home care and nursing home care sectors establish that wages start at the minimum wage. Therefore, informal caregivers, who typically have no qualification and no/limited experience in the care sector, could likely be replaced by workers at the minimum wage.

Nonetheless, it could be informative to compare our estimate with an alternative value, based on the average wage rate in the sector. Unfortunately, such a rate is not available. Because of the heterogeneity of providers, statistics on wages are scattered [9].^9^ Still, we propose here two alternative computations, based on information available on market wages. With a total of 185.8 million informal hours per month, using a rate of $\text{\EUR}$12.63 (based on the median wage rate in the home care sector)^10^ yields a yearly value of informal care of $\text{\EUR}$28.2 billion (16% higher than our baseline estimate); using a rate of $\text{\EUR}$14.3 (based on the average wage rate for over-the-counter workers)^11^ yields a total value of informal care of $\text{\EUR}$31.9 billion euros (32% higher than the baseline).

If we would choose to value informal care based on the average wage in the care sector, these two alternative values would be over-estimations. The first value relies on the median wage in the home care sector, which is expected to be higher than the average wage, due to a large share of home care workers being at the minimum wage [1]. The second case relies on the average rate of over-the-counter workers; those represent about two thirds of the home care workers, but are on average better paid than the other workers (employees of home care organizations).

Our baseline estimation is congruent with available evidence showing the quantitative importance of informal care within long-term care systems. In France, a study looking specifically at the long-term care costs incurred by patients with Alzheimer’s disease has estimated that 70–76% of costs were made of informal care [20]. A recent report by the European Union [16] shows that the value of informal care in France represents about twice that of formal care: 5.0% of GDP against 2.4%, in the basis scenario and using the proxy good method.^12^ The finding that informal care makes for most of total long-term care costs is observed in a majority of the European countries. There is evidence of a North–South gradient, consistent with [4] that shows that informal care represents up 22% of care hours received by older adults in Northern Europe, 81% in Southern Europe, 43% in Continental Europe (which includes France), and 54% in the United States.

Our study adds to this literature by pinpointing the value of the care provided to older adults specifically, which contributes to the debate on the expected costs associated with an ageing population. It is most closely related to [35], who estimate the value of informal care provided to community-dwelling older adults in France. Based on the HID survey (1999–2000), the authors estimate informal care value to reach 6.6 billion 1999 euros (equivalent to 8.2 billion 2015 euros). Our estimation is much higher, both in absolute value (22.9 vs 8.2) and as a share of GDP (1.1% vs 0.47%). This difference, which is further explored in Appendix F, may come from two main sources. First, the parameters (e.g. population size, labor cost etc.) entering the computations may have changed between 1999 and 2016; in particular, the number of the 60+ has increased. Second, there are methodological differences: we use a broader definition of activity restrictions and we observe a volume of care declared by individuals, while [35] impute a volume of care to each respondent (depending on the ADLs/IADLs they have difficulties with).

### Policy implications

This work is policy-relevant in several ways. First, to design an efficient and fair long-term care system, policymakers need to understand the mechanisms explaining care arrangements. We show that the average 60+ with activity restrictions in the community receives much more informal care than the average nursing home resident. Thus, policies promoting aging in place implies shifting additional weight onto relatives. In cases when relatives are not available or not willing to help, policies promoting aging in place could also result in unmet care needs. In addition, our results dismiss the idea of a complete eviction of informal care by institutional care. This could be due either to the preferences of individuals or to shortage in the staff capacity of nursing homes. Caregiving support measures should then also consider informal care provided in nursing homes.

Second, policymakers should take into account the total and relative costs of long-term care services, including non-monetary costs [12]. The monetary value of informal care received by the French aged 60 and older is found to amount to 24.2 billion euros in 2015–2016, or about 1.1% of the Gross Domestic Product (GDP). Given our methodological choices, this value is meant to be a lower bound. Comparing it to the cost of formal LTC services, estimated by the Ministry of Health to $\text{\EUR}$10.5 bn [5], we estimate the value of informal care to be more than twice as large as these formal care costs. Without informal care, the Ministry of Health estimates that 21% of the costs associated to LTC are borne privately by individuals and their relatives ($\text{\EUR}$2.1 bn, over a total of $\text{\EUR}$10.5 bn). Taking the $\text{\EUR}$24.2 bn of informal care into account in the costs borne by households would change this value to reach 76% of the LTC costs. Thus, only a limited proportion of the costs associated with LTC provision is shared collectively.

### Limitations and extensions

Our results should be interpreted bearing in mind some data-related and method-related limitations. Regarding the data, we use the CARE survey implemented in 2015, before the French 2016 reform on LTC.^13^ This reform consisted in a moderate increase in the subsidies towards formal care and a strengthening the support to informal caregivers. Given that the reform was a relatively minor one - it merely reinforced the previous objectives of the French LTC system -, we do not expect a major shift on informal care trends after this reform.

In this survey, we use information on informal care from the CARE survey that results from the respondents’ answers to the questionnaire or that of a proxy respondents. It may come along with measurement errors. Indeed, responses of respondents might be biased in their assessment of who provides care among relatives and the volume of care provided [8, 15]. Moreover, some individuals are not able, or not willing, to quantify precisely the volume of care provided by one or several of their caregivers. Finally, the use of proxy respondents might be associated with noise in the estimation of volume provided, since provider and recipient reports are not necessarily the same [40]. Appendix B.2 explores the difference between caregiver and recipient declaration on volume on a selected subsample of caregiver-recipient for whom both information are available. It shows that caregivers tend to declare slightly more informal care hours than recipients in our data. Using recipient declaration is then consistent with our conservative approach. Appendix E further explores the correlation between respondents’ characteristics and the presence of a proxy respondent. It shows that the presence of a proxy respondent, for informal care questions or for other modules, is mainly related to age and health issues as well as to the availability of a partner.

The proxy good method is associated with a number of assumptions. Regarding the measure of care volume, we should ideally distinguish between the tasks that would have been performed by a relative even without activity restrictions, from the extra tasks performed because the respondent has a care need. The distinction is especially blurry for co-residing informal caregivers. This may play towards an over-estimation of the volume of informal care strictly speaking, since we might take into account care that would have been performed even without activity limitations. In the same vein, the proxy good method requires that time simultaneously spent on several activities is not double counted (e.g. a caregiver does the cooking while also taking care of the laundry). As care time is reported globally for each caregiver (rather than per task) in CARE, our estimations are unlikely to suffer from double-counting.

The proxy good method implicitly assumes that informal care and formal care are perfect substitutes [41]. In this perspective, a formal caregiver is not more efficient at providing care than an informal caregiver, nor does she/he provide better care quality (or vice-versa). Moreover, a unique wage rate is used to value informal care. In practice, some tasks might be performed by skilled caregivers, associated with a higher wage rate. This could not be addressed with our data, which provide a global volume per caregiver and not per task. Finally, our valuation method is partial and focuses only on the volume of care. In particular, it leaves in the shadow the economic costs incurred by caregivers [30], and the (dis-)utility that caregivers and care recipients could derive from the care being provided informally rather than by professional workers. There is evidence that caregivers derive utility from the very process of providing care to a relative (*process utility*), and from seeing their relative appropriately cared for (*altruistic preferences*). On the other hand, the existence of a caregiving burden is now widely acknowledged. It is difficult to conjecture how these effects may weigh on our estimates on balance [13, 24].

In the econometric analysis, we have used the standard, linear Oaxaca-Blinder decomposition. Extensions for non-linear models have been proposed [7]. Given that our outcome (informal care receipt) is binary, we have tested the robustness of our results to a decomposition based on a binary regression. The results, in terms of the contributions of endowments, of coefficients and the interaction term, are quantitatively extremely similar to the estimates derived with the linear probability model.^14^

## Conclusion

This paper provides a comprehensive approach of informal care for the activities of daily living for the 60+ in France, taking into account both individuals living in the community and nursing home residents. We provide a global assessment of the importance and value of informal care provided by relatives to the 60+ and find that a total of 186 millions hours are provided informally each month, mainly in the community (95%). Using the proxy good method, informal care hours represent at minimum 24.2 billion euros per year in 2015/2016, equivalent to 1.1% of GDP. Investigating into the determinants of informal care provision, we find that divergent mechanisms are at play in the determination of informal care receipt across the two settings. It calls for more quantitative research on informal care receipt in nursing homes, which has been under-explored relative to informal care provision to the community-dwelling individuals.

Our findings have two policy implications. First, we highlight the mechanisms explaining care arrangements in both settings. Given our results, policies promoting aging in place thus implies putting extra weight on relatives. At the same time, we show that there is not a complete eviction of informal care in nursing homes. Respite and caregiver support policies should then not only target the relatives of community-dwelling individuals. Overall, when comparing residential care versus aging-in-place, it cannot simply be assumed that professional caregiving within nursing homes replaces all care provided at home (including informal care) after admission. Second, policymakers need to take into account the total and relative costs of LTC services, including non-monetary costs [12]. This is particularly important when comparing the cost of residential and at-home settings. We estimate that households bear 76% of the direct costs associated with LTC provision. The indirect effects of informal care such as reduced labor force participation and adverse health effects for caregivers [6, 11], whose estimation lies beyond the scope of this study, would arguably increase the economic costs of informal care. In other words, even in a country with an extensive Welfare State, the economic costs of old-age disability are only to a limited extent borne collectively. The ageing of society is likely to result in a growing pressure on relatives. Whether this finding is in line with societal preferences is an essentially normative question, which should be addressed in any reform of the long-term care system.

### Supplementary Information

Below is the link to the electronic supplementary material.

Supplementary file1 (PDF 558 KB)

## Footnotes

1 In France, assisted living facilities are called either ‘foyer-logement’ or EHPA (*Etablissement d’hébergement pour personnes âgées*). They are targeted to the 60+ fully autonomous or with only mild functional limitations. There are also two types of nursing homes: EHPADs (*Etablissement d’hébergement pour personnes âgées dépendantes*) and USLDs (*Unités de soins de longue durée)*, the latter ones being part of a hospital and providing more specialized and intensive medical care.

2 Appendix B.2 provides descriptive statistics on informal care volumes as reported at the caregiver level and Appendix B.3 provides a comparison of censored and uncensored distribution of volumes. Situations in which a volume higher than 12 h per day is observed are virtually non-existent for nursing home residents, and fairly rare for caregivers of at-home respondents. Such situations may indicate very high care needs, but also reporting errors.

3 A monetary quantification of the financial and material support is feasible using the CARE data, and can be related to the theoretical and empirical economic literature that has investigated into inter-generational transfers. However, the scope of such an exercise is different from the aim of our study, which focuses on in-kind support and the provision of care.

4 We consider the income at the household level and use the OECD-modified scale [23]. Information on income comes from administrative data linked with the survey.

5 The labor cost is the sum of the gross wage paid to the workers and the social security contributions and taxes levied on employers.

6 Computations of authors, using the gross minimum wage of $\text{\EUR}$9.61 for 2015 (source: [26]) and including effective social security contributions (source: [25]). Several rates exist for social security contributions; we have chosen the lowest rate (12.80%) applying to small units (less than 11 employees).

7 [2] assess the extent to which such differences can be explained by compositional differences in the old-age population across the two countries, or by individual and family characteristics having different effects on the probability of care receipt in the two countries.

8 In Appendix D, Table D.3 presents the same estimates and additionally provides the estimates obtained on the pooled sample, i.e. the determinants of informal care receipt in the whole disabled 60+ population in France. Given that the model is estimated by OLS, the estimates on the pooled sample are a weighted average of those on community-dwellers and nursing home residents.

9 On top of the distinction between the home care sector and the nursing home care sector, work conditions differ depending on whether the worker is self-employed or an employee, and whether the organization is public, not-for-profit or for-profit.

10 [1] provides a median monthly net salary of $\text{\EUR}$1,250, equivalent to a gross hourly wage of $\text{\EUR}$11.2, which we inflate by 12.8% to approximate employer’s social security contributions.

11 [31] provides a net hourly salary of $\text{\EUR}$9.76, equivalent to a gross wage of $\text{\EUR}$12.68, which we inflate by 12.8% to get to the unit labor cost.

12 While we focus on care received by the 60+, [16] looks at *all* informal care provided; further, this report provides a range of estimates for the value of informal care, based either on the proxy good or the opportunity cost method.

13 *Loi relative à l’adaptation de la société au vieillissement* – Law for the adaptation of society to aging.

14 We have used the command nldecompose. Results available on demand.
