# Supplementary material for: Informal care at old age at home and in nursing homes: determinants and economic value
Source: Eur J Health Econ. 2023 Jun 10;25(3):497–511. doi: 10.1007/s10198-023-01601-x (PMC10972991; doi:10.1007/s10198-023-01601-x)
Supplement: Supplementary file 1 — Supplementary file1 (PDF 558 KB) [file 10198_2023_1601_MOESM1_ESM.pdf]

Supplementary Material.

Informal care at old age at home and in nursing homes: determinants and economic value

Quitterie Roquebert* and Marianne Tenand†

This document provides Supplementary Material to the publication:
Roquebert, Q. and Tenand, M. (2023). "Informal care at old age at home and in nursing homes: determinants and economic value", *European Journal of Health Economics.*

*Université de Strasbourg, Université de Lorraine, CNRS, BETA, 67000, Strasbourg, France. E-mail: roquebert@unistra.fr

†Erasmus School of Health Policy & Management (ESHPM), Erasmus Centre for Health Economics Rotterdam (EsCHER), Erasmus University Rotterdam (EUR) & the Netherlands Bureau for Economic Policy Analysis (CPB), The Hague, The Netherlands. E-mail: tenand@eshpm.eur.nl

Contents

A Additional information on data **3**

B Additional descriptive statistics **4**

B.1 Descriptive statistics on ADL and IADL in the reference population 4

B.2 Descriptive statistics at the caregiver level 6

B.3 Informal care volume with and without censoring 9

C Decomposition approach **11**

C.1 Framework: the Oaxaca decomposition 11

C.2 Interpretation and extensions 12

C.3 Implementation 13

D OLS estimations of the determinants of informal care receipt **14**

E Results on proxy responses **16**

F Differences with previous estimates in France **20**

A Additional information on data

The sampling procedures were defined so as to get representative samples of both the population living at home (CARE-M) and the population living in nursing homes (CARE-I).

Regarding CARE-M, a preliminary survey, “Vie quotidienne et santé” (VQS), was conducted on 210,000 households to retrieve information on health and organization of daily life (informal and formal care) through 21 questions. Each VQS respondent was classified into one of four health groups (’VQS group’). The health groups were constructed using information on limitations and restrictions with ADLs or IADLs. The response rate of VQS survey was about 57% [1]. This preliminary survey was used to draw a sample of individuals aged 60 and more for the CARE-M survey. The CARE sample was stratified by local areas (departments, corresponding to NUTS 3 level, 96 units in France) and VQS groups: it over-sampled individuals with poor health or limitations, so as to achieve more statistical precision for analyses focusing on such respondents. Survey weights are however available to make statistics representative of the whole national population. The survey was collected through interviews at home.

Regarding CARE-I, a sample of long-term care institutions was first drawn and surveyed in order to and retrieve the list of residents. Residential long-term care settings include non-medicalized and medicalized nursing homes (respectively EHPA and EHPAD) as well as the long-term care units of hospitals (USLD).[1] In a second step, a sample of permanent residents was drawn within each nursing home. General information about the nursing home and some individual information about the residents that were surveyed (e.g. long-term care transfers received) was collected through a questionnaire at the level of the nursing home (Questionnaire Etablissements). A second questionnaire, containing most of the variables we exploit in our analysis, was administered to the selected residents or to proxy respondent (Questionnaire Seniors). 3,262 respondents from 616 nursing homes participated into the survey.

[1]For the sake of simplicity, we use the term of ‘nursing homes’ for these 3 types of residential care settings.

B Additional descriptive statistics

B.1 Descriptive statistics on ADL and IADL in the reference population

Table B.1 shows the frequency of ADL and IADL limitations in the reference population (living at home and in nursing home). Individuals with missing values on a variable are not taken into account for the computation of frequencies for this variable. Missing values are marginal for individuals living in the community (0 to 34 missing values at most for the variable "Taking transportation"). In nursing homes, missing values are more frequent for some variables due to the structure of the survey. Indeed, there were filters in questions relating to some of the IADLs: in the institution-level questionnaire, it was asked whether residents were allowed to (i) do the grocery, (ii) do the domestic chores, (iii) prepare meals, (iv) manage medication and (v) move around alone. Within institutions in which one or several of these activities were typically not allowed for residents, the questions about their ability to perform these tasks were not asked. By default, the individuals living in nursing homes with such filters are not regarded as having limitations for activities when the question was not asked.

Table B.1: Information on ADL and IADL in the reference population

	(1) At home	(2) Nursing home	(3) Entire population
ADL			
Grooming	30.3	80.6	35.9
Dressing and undressing	30.8	74.5	35.6
Using toilets	8.9	59.6	14.5
Cutting food	15.0	57.7	19.7
Eating and drinking	5.8	33.5	8.8
Transferring from bed	20.2	62.5	24.9
Transferring from a chair	24.5	59.5	28.4
IADL			
Moving in the place where one lives	19.2	60.9	23.8
Doing housework	60.9	94.3	63.4
Doing administrative tasks	55.9	94.1	60.1
Doing grocery shopping	54.1	94.0	58.2
Preparing meals	28.1	94.8	33.0
Taking medication	15.8	76.1	20.4
Using a phone	13.5	68.2	19.5
Going outside	31.3	87.5	37.5
Using transportation	43.2	93.5	48.8
Finding one's way outside	21.1	83.8	28.0
Number of observations in the reference population	6889	3161	10050

SOURCES: CARE-M (2015), CARE-I (2016).

SAMPLES: French 60+ population living at home or in a nursing home.

NOTES: Percentages of population are computed taking into account survey weights and not taking into account missing values. The differences between the sample of individuals living in nursing homes and individuals living at home are all significant at the 1% level (Student test).

B.2 Descriptive statistics at the caregiver level

Extreme values in informal care at the caregiver level

Our measure of informal care receipt at the *respondent* level is constructed using care volume declared at the *caregiver* level. We therefore investigated the occurrence of extreme values at the caregiver level.

Table B.2 reports statistics on the volume of care reported for caregivers in the community and for nursing home residents. No caregiver has a volume higher than an equivalent of 24 hours a day (Column (1)). However, the share of caregivers for which a very higher number of hours is reported (higher than 16 hours/day) reaches 2.8% at home, and 8.0% when we consider instead a threshold of 8 hours a week. As could be expected, the probability of high care volumes is fairly small among caregivers of nursing home residents (Column (2)).

Table B.2: Information on care volume, at caregiver level.

	At home<br>(1)	In nursing homes<br>(2)
Proportion with non-missing volume	100.0%	81.3%
Proportion with volume>24h/day	0.0%	0.1%
Proportion with volume>16h/day	2.8%	0.1%
Proportion with volume>12h/day	3.6%	0.3%
Proportion with volume>8h/day	8.0%	0.6%
Number of informal caregivers	6,512	3,538

SOURCES: CARE-M (2015), CARE-I (2016).
SAMPLES: Informal caregivers providing help with the activities of daily living (ADLs/IADLs) as reported by the survey respondents with activity restrictions.
NOTES: These statistics are *not* weighted by the survey weights at the respondent level.

Co-residence is a major determinant of reporting an extreme care volume. For caregivers of at-home respondents who co-reside with the respondent, the probability of an extreme volume (> 16 days) equals 7% against less than 2% for non-coresiding caregivers. An extreme value is also more frequent among caregivers who are partners (8% report more than 16 hours a day).

Also among nursing home residents, a high volume generally indicates that the caregiver is the partner of the respondent. In almost all cases, for nursing home residents a partner reports a high volume only if h/she lives with the respondent in the nursing home.

Comparison of caregiver and recipient volumes

In the survey, we have some information on volume directly obtained from caregivers. Indeed, individuals who were declared as caregivers by respondents in CARE-M have been asked to answer a specific survey on caregivers ("CARE-M Aidants"). They have been asked a number of questions regarding the care they provide, in particular they are asked to estimate the volume of care they provide. Among children providing care to individuals having ADLs/IADLs restrictions, 81% responded to the specific survey on caregivers. It gives the opportunity to compare the volume declared by care recipient and caregiver in our data. When both declare a given volume (no missing values nor intervals). The distribution of the difference between recipient volume and caregiver volume is displayed in Figure B.1. We differentiate between the volume declared without censoring and with censoring at 12h per day at caregiver level. Without censoring, the median difference is zero, with a mean at -7. With censoring at 12 hours per day at the caregiver level, the difference is even more reduced (median at 0, mean at -5.5). Overall, on average, recipient tends to declare a lower volume than caregivers. Since we adopt a conservative approach in which we provide a lower bound for informal care value, it thus makes sense to rely on recipient declaration.

Figure B.1: Distribution of the difference between recipient and caregiver declaration on informal care hours received, per month (at-home population)

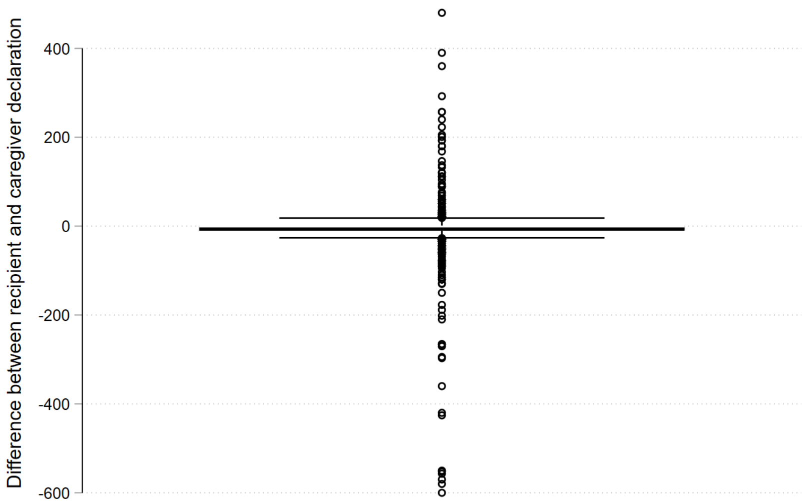

(Upper) Without censoring

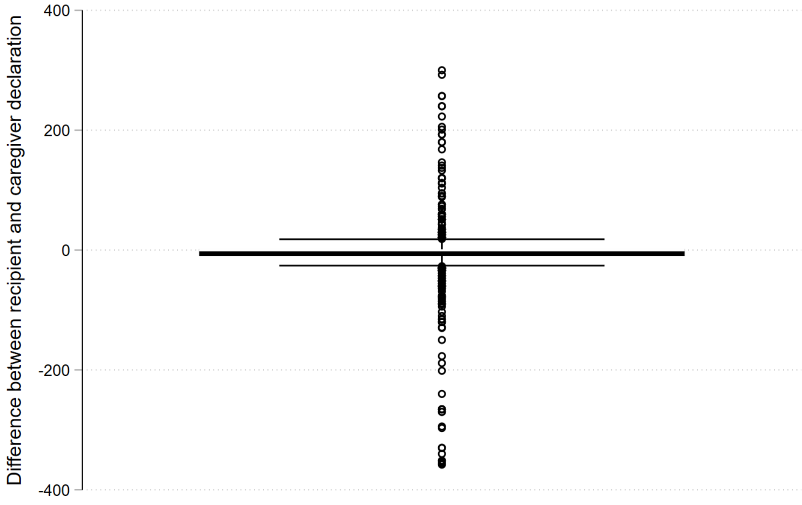

(Bottom) With censoring at 12 hours per day

Sources: CARE-M (2015)
Sample: 1,145 caregivers of a 60+ individual with activity restrictions, living at home, providing informal care for the activities of daily living, with care volume provided both by care recipient and caregiver.
Notes: Censored volumes correspond to volume with censoring (i) at the caregiver level (12 hours per day over 30 days in the month, maximum).

B.3 Informal care volume with and without censoring

The measure of informal care volume we use in the valuation exercise and the comparative analysis of individual determinants of informal care receipt was censored, so as to avoid outliers and reporting errors to weigh excessively on our results.

This section compares the censored and uncensored distributions of informal care volumes. Focusing on informal care volumes for at-home population, we see that both distributions are rather similar, with a median value at 45 hours, and similar percentiles (25% = 16 hours, 75% = 120 hours, 90% = 300 hours). Censoring affects the extreme values (95th percentile = 450 hours without censoring, 360 with censoring; (99th percentile=733 without censorship, 438 hours with censoring; maximum values at 1,560 hours without censoring, 720 hours with censoring), which has consequences for the mean value of the distribution (110 hours without censoring, 95 hours with censoring). Similarly, in nursing homes, the distribution of volumes is close except for the 1% extreme highest values.

Figure B.2: Distribution of the number of informal care hours received, per month, among recipients (censored and uncensored volumes)

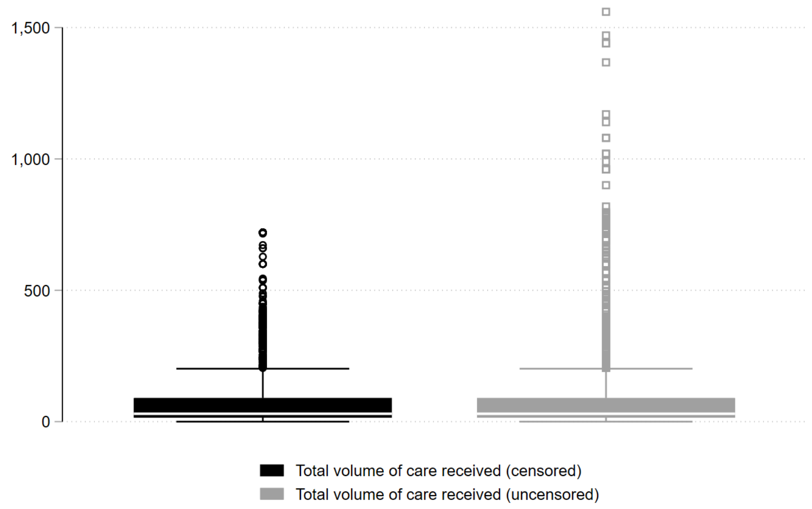

(Upper panel) At-home population.

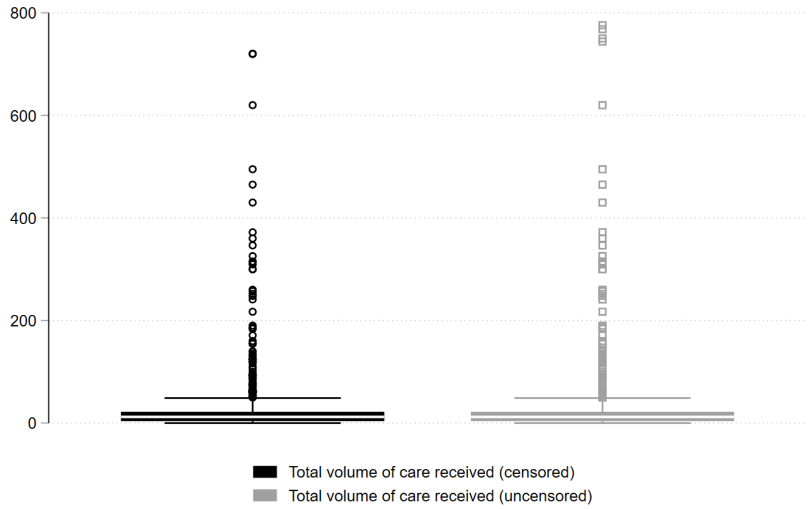

(Bottom panel) Nursing home residents.

Sources: CARE-M (2015), CARE-I (2016).
Samples: French 60+ population, with activity restrictions, living at home (upper) or in an nursing home (bottom), receiving informal care for the activities of daily living.
Notes: Statistics are weighted using the survey weights. Censored volumes correspond to volume with censoring (i) at the caregiver level (12 hours per day over 30 days in the month, maximum) and (ii) at the respondent level (24 hours per day over 30 days in the month, maximum). Uncensored volumes correspond to volume without any censoring.

C Decomposition approach

C.1 Framework: the Oaxaca decomposition

We adopt here the framework initially developed by [5]. A useful overview of this approach is proposed by [6].

We denote y_i the informal care received by individual i. For individuals living in institutions, we assume that y_i can be expressed as:

$$y_i = \beta^{inst} X_i + u_i^{inst} \tag{C.1}$$

where $X_i = (1, x_i^1, ..., x_i^J)$ is a vector including J covariates and β^{inst} a vector of parameters including an intercept. u_i^{inst} captures the unobserved determinants of informal care receipt in institutions.

Similarly, for individuals living in the community, we assume that y_i can be expressed as:

$$y_i = \beta^{home} X_i + u_i^{home} \tag{C.2}$$

where β^{home} a vector of parameters including an intercept and u_i^{home} captures the unobserved determinants of informal care receipt at home.

Using Equations (C.1) and (C.2), we can express mean informal care in both populations as a function of the parameter estimates $\hat{\beta}$ and of the covariates, and the gap between mean informal care receipt in institution (denoted $\bar{y}^{inst}$) and mean informal care receipt at home ($\bar{y}^{home}$) as:

$$\bar{y}^{inst} - \bar{y}^{home} = \hat{\beta}^{inst} \bar{X}^{inst} - \hat{\beta}^{home} \bar{X}^{home} \tag{C.3}$$

where $\bar{X}^{inst}$ (resp. $\bar{X}^{home}$) denotes the population average of covariates for the institutionalized population (resp. for the non-institutionalized population).

Going one step further, we can decompose the gap in average outcomes into two parts. One first possible Oaxaca decomposition is:

$$\bar{y}^{inst} - \bar{y}^{home} = \hat{\beta}_0^{inst} + \sum_{j=1}^{J} \hat{\beta}_j^{inst} \bar{x}^{j,inst} - \hat{\beta}_0^{home} - \sum_{j=1}^{J} \hat{\beta}_j^{home} \bar{x}^{j,home} \tag{C.4}$$

$$= \left(\hat{\beta}_0^{inst} - \hat{\beta}_0^{home}\right) + \sum_{j=1}^{J} \bar{x}^{j,home} \left(\hat{\beta}_j^{inst} - \hat{\beta}_j^{home}\right)) \qquad + \sum_{j=1}^{J} \hat{\beta}_j^{inst} \left(\bar{x}^{j,inst} - \bar{x}^{j,home}\right) \tag{C.5}$$

$$= \Delta_1^U + \Delta_1^E \tag{C.6}$$

Δ_1^E equals $\sum_{j=1}^J \hat{\beta}_j^{inst}\big(\bar{x}^{j,inst} - \bar{x}^{j,home}\big)$ and is the part of the gap in informal care receipt that can be explained by compositional differences between the institutionalized population and the non-institutionalized elderly, scaled up by the association between covariates and informal care receipt among the institutionalized elderly. Δ_1^U equals $\big(\hat{\beta}_0^{inst} - \hat{\beta}_0^{home}\big) + \sum_{j=1}^J \bar{x}^{j,home}\big(\hat{\beta}_j^{inst} - \hat{\beta}_j^{home}\big)\big)$ and corresponds to the part of the gap in informal care receipt that cannot be explained by differences in the observed determinants of care. This part can be attributed either to differences in the association between a given covariate and care receipt across home and institutional settings (i.e. the difference $\big(\hat{\beta}_j^{inst} - \hat{\beta}_j^{home}\big)\big)$, scaled up by the population mean of the covariates in the home care setting, or to systematic residual differences (captured by the difference in the intercepts, $\big(\hat{\beta}_0^{inst} - \hat{\beta}_0^{home}\big)\big)$.

Put it differently, Δ_1^E would be equal to zero if the composition of the non-institutionalized population were the same as the composition of the institutionalized population. Δ_1^U is the residual gap.

Note that the gap in informal care receipt across the two settings can also be written as:

$$\bar{y}^{inst} - \bar{y}^{home} = \big(\hat{\beta}_0^{inst} - \hat{\beta}_0^{home}\big) + \sum_{j=1}^{J} \bar{x}^{j,inst}\big(\hat{\beta}_j^{inst} - \hat{\beta}_j^{home}\big)\big) \quad + \sum_{j=1}^{J} \hat{\beta}_j^{home}\big(\bar{x}^{j,inst} - \bar{x}^{j,home}\big) \tag{C.7}$$

$$= \Delta_2^U + \Delta_2^E \tag{C.8}$$

where Δ_2^U and Δ_2^E are, respectively, the unexplained part and the explained part of the gap in informal care receipt in this second Oaxaca decomposition.

C.2 Interpretation and extensions

The difference between the two decompositions is which setting (home or institution) is taken as the reference for the scaling of the difference in the β's and of the difference in the x's. In the general case, the two decomposition formulas will *not* deliver the same results. This is because the first decomposition implicitly attributes the interaction between the compositional difference and the difference in coefficients in the unexplained part (Δ_1^U), while the second decomposition attributes it to the explained part (Δ_2^E) (see [6]).

Several extensions of the [5] decomposition have been provided. See e.g. [2, 4, 9].

C.3 Implementation

The user command `decompose` can be used to implement the Oaxaca and related decompositions in Stata.

As we use survey data with unequal sampling probability and non-response correction, we must take into the survey weights to derive average informal care receipt and the average values of covariates among the non-institutionalized and the institutionalized populations. In addition, we run weighted regressions to estimate coefficients β^{inst} and β^{home}.

To take into account potential correlations of disturbances among individuals living in the same setting, we estimate standard errors clustered at the household level (for the elderly in the community) or institution level (for nursing home residents).

D OLS estimations of the determinants of informal care receipt

Table D.1: Determinants of informal care receipt

Outcome:	*Receives informal care*		
	At home	In nursing homes	All
	(1)	(2)	(3)
Woman	0.037	0.017	0.033
	(0.023)	(0.018)	(0.021)
Age: 60-74	0.036	-0.114***	0.021
	(0.026)	(0.035)	(0.025)
Age: 75-84	Ref.	Ref.	Ref.
Age: 85-89	0.061**	0.050**	0.072***
	(0.027)	(0.023)	(0.024)
Age: 90-94	0.141***	0.072***	0.145***
	(0.033)	(0.022)	(0.027)
Age $\geq$ 95	0.102**	0.064**	0.118***
	(0.046)	(0.030)	(0.033)
Married or civil union	Ref.	Ref.	Ref.
Widow	0.080***	-0.072***	0.072***
	(0.025)	(0.021)	(0.023)
Single or divorced	-0.046	-0.150***	-0.053**
	(0.030)	(0.028)	(0.027)
Children: none	-0.011	-0.229***	-0.046*
	(0.030)	(0.024)	(0.026)
Sister(s) or brother(s) alive	-0.007	0.059***	0.003
	(0.022)	(0.015)	(0.019)
Diploma: none	0.064**	-0.038**	0.055**
	(0.025)	(0.019)	(0.022)
Diploma: secondary education	-0.006	-0.008	-0.007
	(0.027)	(0.023)	(0.025)
Diploma: primary education	Ref.	Ref.	Ref.
Diploma: higher education	-0.049	-0.043	-0.046
	(0.042)	(0.039)	(0.039)
Diploma: missing	-0.065	-0.143***	-0.168***
	(0.094)	(0.023)	(0.030)
Income: $\leq$ 14,999	0.012	-0.033*	0.005
	(0.025)	(0.018)	(0.022)
Income:15,000-19,999	Ref.	Ref.	Ref.
Income: 20,000-29,999	-0.043	0.009	-0.039
	(0.027)	(0.020)	(0.025)

Continued on next page

Table D.1 – *Continued from previous page*

Outcome:	*Receives informal care*		
	At home	Nursing home	All
	(1)	(2)	(3)
Income: $\geq$ 30,000	-0.069*	-0.041	-0.068**
	(0.036)	(0.032)	(0.033)
Activity restrictions: moderate	Ref.	Ref.	Ref.
Restrictions: ADL, except those of minimum independence	-0.033	0.047*	-0.021
	(0.022)	(0.027)	(0.021)
Restrictions: ADL on minimum independence	0.007	0.019	-0.010
	(0.033)	(0.029)	(0.025)
Alzheimer's Disease	0.117***	-0.042**	0.048**
	(0.031)	(0.017)	(0.021)
Limitations: cognitive	-0.010	-0.002	-0.005
	(0.027)	(0.035)	(0.026)
Limitations: sensory	0.051**	0.018	0.044**
	(0.022)	(0.018)	(0.020)
Limitations: suppleness, handling	0.016	0.099*	0.016
	(0.039)	(0.060)	(0.038)
Limitations: locomotion, balance	0.104***	0.023	0.110***
	(0.028)	(0.036)	(0.027)
Incontinence	-0.002	-0.014	-0.002
	(0.021)	(0.018)	(0.019)
Self-reported chronic disease or health condition	-0.004	0.011	-0.016
	(0.029)	(0.019)	(0.025)
Subjective health: bad or very bad	0.085***	-0.017	0.074***
	(0.021)	(0.018)	(0.019)
Subjective health: fairly good	Ref.	Ref.	Ref.
Subjective health: good or very good	-0.025	0.014	-0.017
	(0.029)	(0.020)	(0.026)
Proxy on informal care questions	0.207***	0.353***	0.224***
	(0.033)	(0.032)	(0.030)
Proxy on other parts of the questionnaire	0.168***	-0.181***	0.137***
	(0.032)	(0.035)	(0.030)
Constant	0.230***	0.679***	0.261***
	(0.058)	(0.067)	(0.054)
Observations	6889	3161	10050
R^2	0.220	0.252	0.217
Number of clusters	6889.000	613.000	7502.000

SOURCES: CARE-M (2015), CARE-I (2016).

SAMPLES: French 60+ population, with activity restrictions, living at home or in a nursing home.

NOTES: Estimations of linear probability models. Standard errors in parentheses, clustered at the household level (at-home respondents) or at the institution level (institutionalized respondents). * $p < 0.10$, ** $p < 0.05$, *** $p < 0.01$.

E Results on proxy responses

In this Appendix, we explore (i) the individual characteristics correlating with the use of a proxy respondent (hereafter: proxy) and (ii) the correlation between the presence of a proxy and informal care receipt.

Table E.I presents the estimation of the probability to have a proxy on informal care questions (Column 1) or on other modules (Column 2). The probability to have a proxy on informal care questions is correlated to age, with older people being more likely to have a proxy. Individuals with health issues (restrictions, limitations, Alzheimer disease, incontinency, bad subjective health) have also a higher probability to have a proxy, except for people with suppleness/handling limitations. Individuals without partner and with a lower income have a lower probability to have a proxy on informal care questions, while the correlation with education is erratic. The correlates of proxy presence for informal care questions or other modules are similar, except that woman have a lower probability to have a proxy for other modules, and regarding the effect of income. Overall, the presence of proxy is related to age and health issues as well as to the availability of a partner.

Table E.I: Correlates to proxy

Outcome:	*Presence of proxy*	
	For informal care questions	For other modules
	(1)	(2)
Woman	-0.023	-0.043**
	(0.018)	(0.020)
Age: 60-74	-0.055***	-0.050**
	(0.020)	(0.024)
Age: 75-84	Ref.	Ref.
Age: 85-89	0.077***	0.049**
	(0.022)	(0.023)
Age: 90-94	0.121***	0.135***
	(0.028)	(0.026)
Age $\geq$ 95	0.268***	0.272***
	(0.050)	(0.044)
Married or civil union	Ref.	Ref.
Widow	-0.132***	-0.296***
	(0.019)	(0.021)
Single or divorced	-0.165***	-0.367***
	(0.022)	(0.023)
Children: none	-0.021	-0.021
	(0.024)	(0.026)

Sister(s) or brother(s) alive	-0.010	0.013
	(0.017)	(0.018)
Diploma: none	0.093***	0.117***
	(0.021)	(0.022)
Diploma: secondary education	-0.053***	-0.057**
	(0.019)	(0.023)
Diploma: primary education	Ref.	Ref.
Diploma: higher education	-0.052	-0.103***
	(0.034)	(0.038)
Diploma: missing	0.114***	0.216***
	(0.029)	(0.024)
Income: $\leq$ 14,999	0.041**	0.026
	(0.020)	(0.022)
Income:15,000-19,999	Ref.	Ref.
Income: 20,000-29,999	-0.007	-0.035
	(0.020)	(0.023)
Income: $\geq$ 30,000	0.003	0.001
	(0.026)	(0.031)
Activity restrictions: moderate	Ref.	Ref.
Restrictions: ADL, except those of minimum independence	0.074***	0.040**
	(0.018)	(0.020)
Restrictions: ADL on minimum independence	0.266***	0.209***
	(0.026)	(0.030)
Alzheimer's Disease	0.391***	0.341***
	(0.025)	(0.020)
Limitations: cognitive	0.053***	0.092***
	(0.021)	(0.024)
Limitations: sensory	0.017	0.006
	(0.017)	(0.020)
Limitations: suppleness, handling	-0.061**	-0.101***
	(0.030)	(0.035)
Limitations: locomotion, balance	0.079***	0.058**
	(0.022)	(0.025)
Incontinency	0.048**	0.033*
	(0.019)	(0.020)
Self-reported chronic disease or health condition	0.045**	0.028
	(0.021)	(0.024)
Subjective health: bad or very bad	0.046***	0.015
	(0.018)	(0.018)

Subjective health: fairly good	Ref.	Ref.
Subjective health: good or very good	0.013	-0.007
	(0.022)	(0.026)
Constant	0.164***	0.506***
	(0.044)	(0.049)
Observations	10050	10050
R^2	0.257	0.253
Number of clusters	7502.000	7502.000

SOURCES: CARE-M (2015), CARE-I (2016).

SAMPLES: French 60+ population, with activity restrictions, living at home or in a nursing home.

NOTES: Estimations of linear probability models. Standard errors in parentheses, clustered at the household level (at-home respondents) or at the institution level (institutionalized respondents). * $p < 0.10$, ** $p < 0.05$, *** $p < 0.01$.

In the estimations, the presence of a proxy (Figure E.1) is associated with a substantially higher probability to receive informal care in both settings. However, the presence of a proxy specifically on questions not related to informal care *decreases* the probability of informal care receipt in nursing homes. It might reveal that proxys answering other modules are generally professional caregivers, answering for individuals without informal caregivers.

Figure E.1: Proxy determinants of informal care (extensive margin)

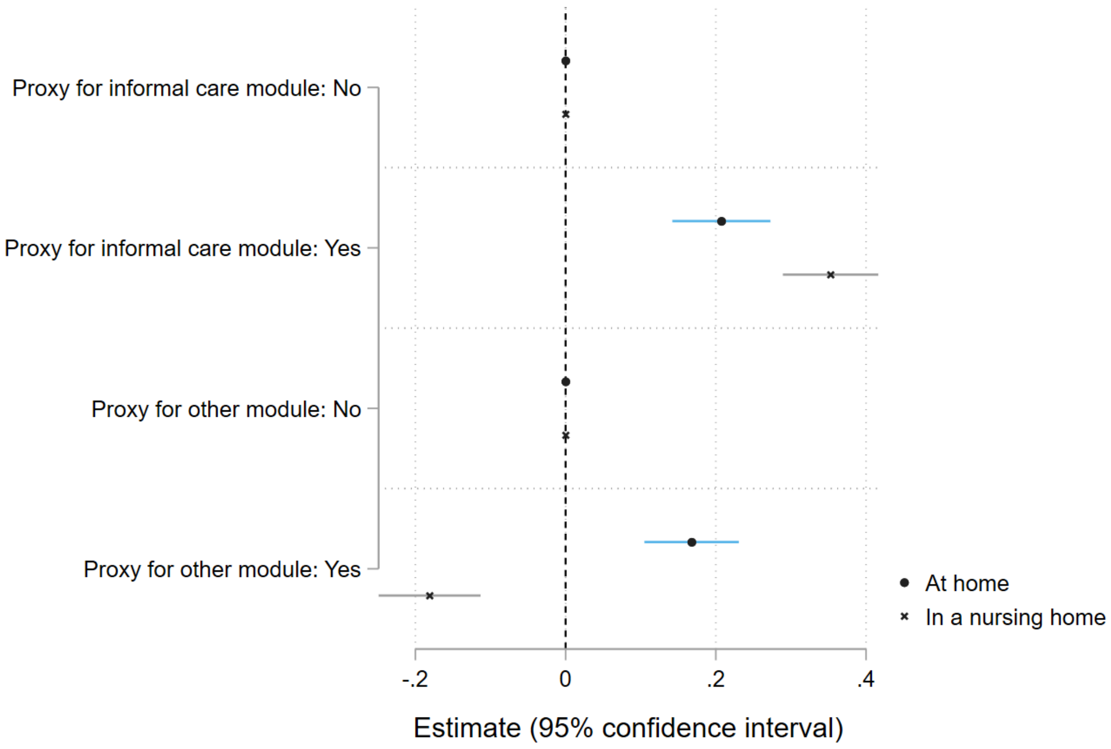

SOURCES: CARE-M (2015), CARE-I (2016).
SAMPLES: French 60+ population, with activity restrictions, living at home or in an nursing home.
NOTES: Coefficients corresponding to the OLS regressions of the probability to receive informal care on socio-demographic, health and proxy variables.

F Differences with previous estimates in France

Despite its conservative features, our estimation is substantially higher than the one previously derived for France by Paraponaris et al (hereafter: PDV). [8]. Based on the HID survey (1999-2000), PDV estimate the value of informal care provided to the 60+ to reach 6.6 billion 1999 euros. In constant euros, our estimation is about 2.8 times higher than that of PDV.[2] Comparing each value with GDP (from 1999 and 2016 respectively), we end up with a value of informal care *as a share of GDP* considerably higher in 2016 (1.1%) compared to 1999 (0.5%).

Formally, the aggregate value of informal care can be computed as follows:

$$Aggregate\ value = A \times B \times C \times D \tag{C.9}$$

Where:

- A is the 60+ population size;
- B is the disability rate (i.e. the share of the population that has activity restrictions), such that $A \times B$ corresponds to the size of the population with activity restrictions;
- C is the average number of hours received by a 60+ older disabled adult;
- D is the valuation rate.

The differences across our estimate and that of PDV may come from each of factors A, B, C and D.

A first explanation relates to demographic change (factor A) . The 60+ population has increased from 20.6 million people in 1999-2000 to 25.1 million in 2016 [3], an increase of about 25%.

A second explanation relates to differences in the epidemiological parameter (B). It may be that the disability rate has changed over time. Or that there are differences in what is defined as 'disability'. As a matter of fact, we use a broader definition of activity restrictions. PDV delimit their study population to individuals who report having *a lot of* difficulties or unable to perform an IADL or ADL without help. By contrast, we consider that individuals face an activity restriction as soon as they report having *some* difficulties in performing an ADL or IADL. Our estimation thus arguably encompasses all 60+ individuals with LTC needs.

[2]Inflation between 1999 and 2016 was of 24.7% in the French economy [3], such that the PDV estimate is equivalent to 8.2 billion 2016 euros.

Third, the average number of informal care hours received by people with activity restrictions may differ between our study and that of PDV (factor C). From a methodological perspective, the quantification of informal care hours differs in both approaches. We use the volume declared by respondents or their proxy. Instead, the survey leveraged by PDV does not contain any information on care volumes. Their methodology rests on the estimation of the number of hours necessary to perform each ADL and IADL as proposed by [7], combined with the type of ADL/IADL relatives provide care as reported in the survey. It is therefore not possible to infer whether and how the number of hours of care *actually* received has changed between 1999 and 2015/2016.

Finally, the valuation rate used for hours of informal care (factor D) may be different. Both PDV and we use the hourly labor cost (gross wage augmented with employer social security contributions and taxes) at the national minimum wage. [8] use a rate of €11.05 per hour in 1999 while we use €10.84 in 2016. Rates are then relatively close, and as a matter of fact the rate is lower in 2015 than in 1999, due to the introduction of large rebates on social security contributions for low wages. Therefore, the rate at which hours of informal care are valued in the two studies can not explain why our estimation is so much higher than PDV.

PDV do not provide the details of B and C, such that we are not able quantify the contribution of each of these factors to the difference between their estimate and ours.

References

[1] A. Carrère, O. Haag, and N. Soullier. Échantillonnage des enquêtes VQS 2014 et CARE-ménages-2015. Dossiers de la Drees 43, Drees, Oct. 2019.

[2] J. Cotton. On the decomposition of wage differentials. *The review of economics and statistics*, pages 236–243, 1988.

[3] Insee. *Tableaux de l'Économie Française. Édition 2019.* Institut national de la statistique et des études économiques, mar 2019.

[4] D. Neumark. Employers' discriminatory behavior and the estimation of wage discrimination. *Journal of Human resources*, pages 279–295, 1988.

[5] R. Oaxaca. Male-female wage differentials in urban labor markets. *International economic review*, pages 693–709, 1973.

[6] O. O'Donnell, E. van Doorslaer, A. Wagstaff, and M. Lindelow. *Analyzing health equity using household survey data: a guide to techniques and their implementation.* The World Bank, 2007.

[7] R. Pampalon, A. Colvez, and D. Bucquet. Etablissement d'une table de passage de la dépendance des personnes âgées au besoin d'aide à domicile. *Revue d'Épidémiologie et de Santé Publique*, 39(3):263–273, 1991.

[8] A. Paraponaris, B. Davin, and P. Verger. Formal and informal care for disabled elderly living in the community: an appraisal of French care composition and costs. *The European Journal of Health Economics*, 13(3):327–336, June 2012.

[9] C. W. Reimers. Labor market discrimination against hispanic and black men. *The Review of Economics and Statistics*, pages 570–579, 1983.
